# Green‐Processed Non‐Fullerene Organic Solar Cells Based on Y‐Series Acceptors

**DOI:** 10.1002/advs.202303842

**Published:** 2023-08-01

**Authors:** Chunhui Liu, Jinfeng Liu, Xiaopeng Duan, Yanming Sun

**Affiliations:** ^1^ School of Chemistry Beihang University Beijing 100191 P. R. China

**Keywords:** efficiency, green‐processed solvents, Hansen solubility parameters, organic solar cells, Y‐series acceptors

## Abstract

The development of environmentally friendly and sustainable processes for the production of high‐performance organic solar cells (OSCs) has become a critical research area. Currently, Y‐series electron acceptors are widely used in high‐performance OSCs, achieving power conversion efficiencies above 19%. However, these acceptors have large fused conjugated backbones that are well‐soluble in halogenated solvents, such as chloroform and chlorobenzene, but have poor solubility in non‐halogenated green solvents. To overcome this challenge, recent studies have focused on developing green‐processed OSCs that use non‐chlorinated and non‐aromatic solvents to dissolve bulk‐heterojunction photoactive layers based on Y‐series electron acceptors, enabling environmentally friendly fabrication. In this comprehensive review, an overview of recent progress in green‐processed OSCs based on Y‐series acceptors is provided, covering the determination of Hansen solubility parameters, the use of non‐chlorinated solvents, and the dispersion of conjugated nanoparticles in water/alcohol. It is hoped that the timely review will inspire researchers to develop new ideas and approaches in this important field, ultimately leading to the practical application of OSCs.

## Graphical abstract

Green‐processed organic solar cells based on Y‐series electron acceptors have been comprehensively reviewed in this article, focusing on non‐halogenated solvents from aromatic, non‐aromatic, and water/alcohol solvents for solution‐processing photoactive layers. The key problems and the possible way to tackle these issues have also been raised to further improve the photovoltaic performance of this kind of cells.

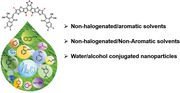

## 1 Introduction

Organic solar cells (OSCs) represent the third generation of solar cells that utilize organic conjugated materials as the photoactive layer. These flexible electronic devices hold significant potential for a wide range of applications, including internet of things, indoor photovoltaics, greenhouses, and smart windows.^[^ ^1^ ^]^ OSCs have demonstrated remarkable progress in recent years, achieving power conversion efficiencies (PCEs) over 19%,^[^ ^2^, ^3^, ^4^, ^5^ ^]^ and exhibiting excellent thermal and light stability with lifetimes exceeding 10 years.^[^ ^6^ ^]^ Additionally, OSCs have demonstrated high mechanical stability under bending tests, making them ideal for use in flexible devices.^[^ ^7^, ^8^ ^]^ High throughput printing techniques have also enabled the production of large‐area devices.^[^ ^9^ ^]^ These favorable characteristics underscore the potential of OSCs for practical applications.

The photoactive layer of OSCs typically comprises two conjugated materials, an electron donor and an electron acceptor. They are usually dissolved in organic solvents such as chloroform (CF), chlorobenzene (CB), and *ortho*‐dichlorobenzene (*o*‐DCB). The donor and acceptor materials are mixed together in solution and deposited onto substrates using spin‐coating for small‐area devices or printing techniques for large‐area devices, resulting in the formation of photoactive layer. The optimization of the photoactive layer, particularly the microphase separation between the donor and acceptor materials, is critical to achieving high photovoltaic performance.^[^ ^10^, ^11^ ^]^

The solution‐process of photoactive layer is hindered by the use of organic solvents containing halogen and aromatic compounds, which pose a significant threat to both human health and the environment. Thus the use of these solvents in industrial fabrication has been restricted. Therefore, the development of non‐halogenated and non‐aromatic solvents for the fabrication of photoactive layers is crucially important. This topic has garnered considerable attention, from the fullerene‐based OSCs era to rylene diimides‐based OSCs, and more recently, near‐infrared electron acceptors. As a result, numerous reviews have focused on this topic.^[^ ^12^, ^13^, ^14^, ^15^, ^16^, ^17^ ^]^

Y‐series acceptors with the A‐D‐A'‐D‐A configuration and a fused core have been successfully utilized in OSCs, significantly improving the PCEs.^[^ ^18^, ^19^, ^20^ ^]^ However, their large conjugated backbones and high crystallinity with 3D networks result in poor solubility in green solvents.^[^ ^21^, ^22^ ^]^ Consequently, numerous strategies have been developed to address the solubility issues in these solvents, ranging from chemical structure design to device optimization, to achieve optimized microphase separation in bulk‐heterojunction (BHJ) thin films. This review provides an overview of these strategies, specifically non‐halogenated aromatic solvents, non‐halogenated/non‐aromatic solvents, and water/alcohol‐based solutions of conjugated nanoparticles, to realize green‐solution processed OSCs based on Y‐series acceptors. In the final section, potential solutions to remaining issues are proposed. This timely review highlights the importance of this field and aims to inspire new ideas toward the development of high‐performance green solution‐processed OSCs.

## 2 Hansen Solubility Parameters (HSPs)

The solubility parameter approach is a powerful tool for determining the physical properties of solute and solvent. It can be used to find the solvent to dissolve conjugated materials. HSP divided the solubility parameters into three parts: atomic dispersive (*δ*_D_), permanent dipole (*δ*_P_), and hydrogen bonding (*δ*_H_) parameters, which can be visualized in a 3D coordinate system. Brabec et al. developed a method to determine the HSP of conjugated materials via selecting many solvents to dissolve them, among which were catalyzed as no solvent and good solvent.^[^ ^23^ ^]^ HSPs of all the good solvents were collected into the coordinate system, so that the HSP of conjugated polymers and the PCBM acceptor could be obtained via the HSPiP software.^[^ ^24^ ^]^ Further, other solvents with known HSPs were put into the coordinate system to compare with the HSPs of conjugated materials to determine no or good solvent. With this method, some new solvents could be found as the good solvents, which is the key principle to select green solvents for solution‐processed OSCs.

Since it is a complicate method to select many solvents as the standard to determine the HSPs of conjugated materials, Brabec et al. then developed a binary solvent gradient method to simplify the calculation process.^[^ ^25^ ^]^ Taking poly(3‐hexylthiophene) (P3HT) and PCBM as the example, CB was selected as good solvent. Three no‐solvents, acetone, oleic acid, cyclohexane for P3HT, and acetone, isopropanol, DMSO for PCBM, were selected. By gradually adding the no‐solvent into CB, the solubility of P3HT or PCBM in the cosolvent was determined, so that the Hansen sphere of P3HT or PCBM in the 3D coordinate system could be obtained with the defining solubility limit of 5 mg mL^−1^. Therefore, the HSPs of P3HT and PCBM were obtained, which then can be used to find new solvents.

**Figure 1:**
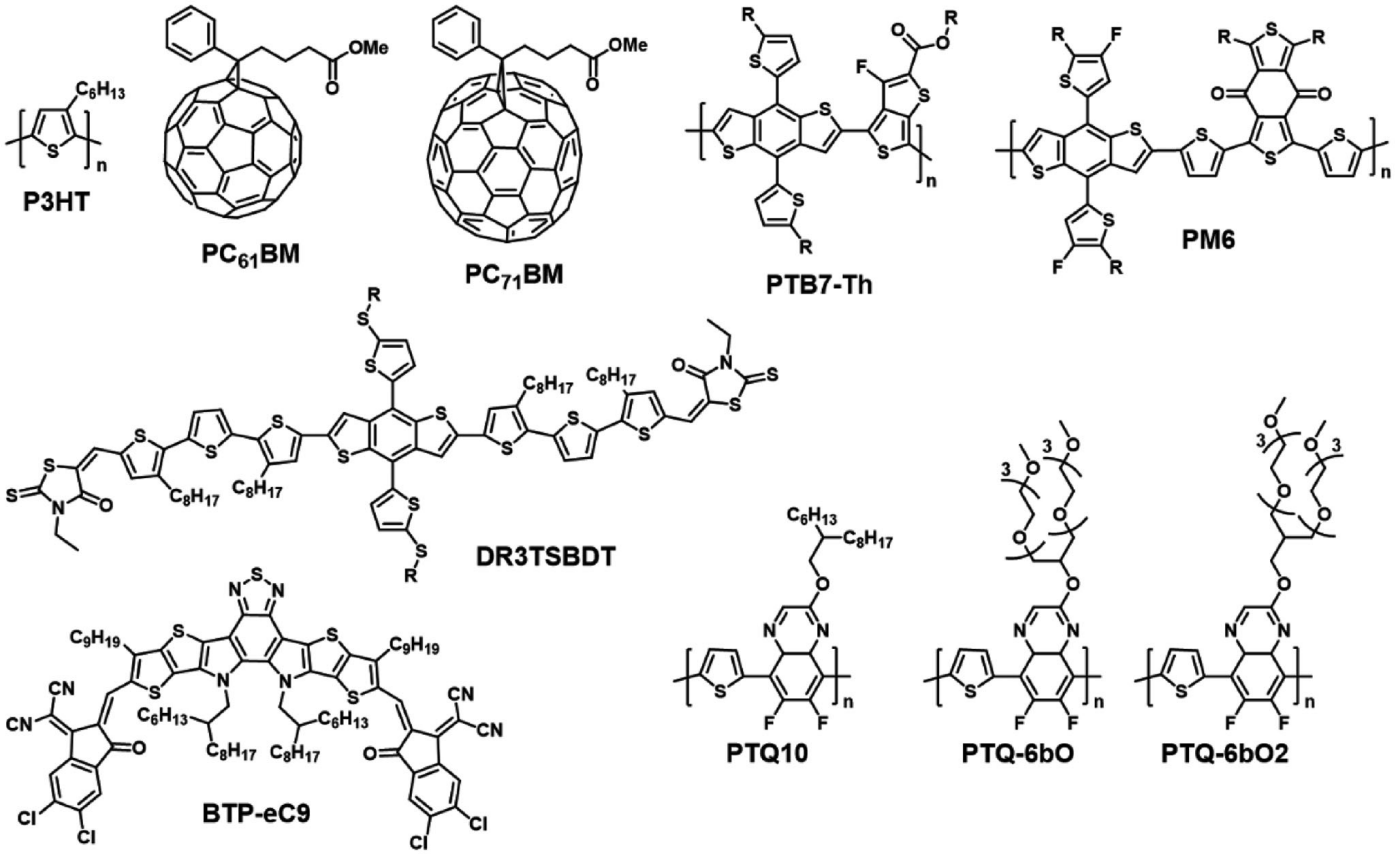
The chemical structures of conjugated materials mentioned in Table 1.

**Table** **1** provides a summary of the HSP and *R*_0_ (representing the radius of the solubility sphere) of conjugated materials from the literature, along with the chemical structures presented in **Figure** **1**. The solubility limit is arbitrary and can affect the HSP, since a higher solubility limit results in a smaller *R*_0_ and less effective solvents. However, replacing alkyl side chains with alkyloxy units can greatly improve the *δ*_P_ and *δ*_H_ values, which allowed PTQ‐6bO and PTQ‐6bO2 polymers to be dissolved in green solvents like alcohol.^[^ ^26^ ^]^ Despite this progress, only a few studies have explored the relationship between chemical structures and HSPs of conjugated materials. More studies in this area can aid in the development of new conjugated materials that are amenable to green processing.

**Table 1:** HSPs of some represent conjugated materials reported in the literatures

Solute	Solubility limit [mg mL^−1^]	*δ*_D_ [MPa^1/2^]	*δ*_P_ [MPa^1/2^]	*δ*_H_ [MPa^1/2^]	*R*_0_ ^a)^ [MPa^1/2^]	Ref.
P3HT	5	18.6	3.4	1.2	1.4	[25]
P3HT	2	18.3	3.9	0.3	2.6	[25]
PC_61_BM	5	19.6	7.1	5.8	4.9	[25]
PC_61_BM	2	19.7	7.4	6.6	5.8	[25]
PC_71_BM	2	19.88	2.85	6.00	–	[27]
PTB7‐Th	2	18.60	2.56	5.71	–	[27]
DR3TSBDT	2	19.88	2.85	6.00	–	[27]
PM6	5	18.4	3.54	2.93	2.5	[28]
BTP‐eC9	5	18.2	2.7	4	3.5	[28]
PTQ10	10	19.2	1.9	3.7	4.7	[26]
PTQ‐6O	10	18.5	3.8	6.1	1.7	[26]
PTQ‐6bO	10	19.5	9.7	7.0	11.9	[26]
PTQ‐6bO2	10	19.5	9.7	7.0	11.9	[26]

^a)^ *R*_0_ (solubility capacity) is defined as the radius of the sphere (HSP of the solute as the central coordinate), representing the maximum distance that enables the arbitrary solubility.

## 3 Non‐Halogenated/Aromatic Solution‐Processed OSCs

When comparing the HSPs of halogenated and non‐halogenated aromatic solvents, there are differences in the *δ*_P_ and *δ*_H_ values. For example, *δ*_D_, *δ*_P_, *δ*_H_ values of CB are 19, 4.3, 2, while for toluene (Tol) and *o*‐xylene (XY), the values are 18, 1.4, 2, and 17.8, 1, 3.1, respectively. Despite these differences, many conjugated polymer donors and Y‐series acceptors are still soluble in these non‐halogenated/aromatic solvents. For instance, XY has similar boiling point to CB (143 °C vs 132 °C), so that XY has been widely used to prepare non‐halogenated OSCs. The good solubility of these non‐halogenated aromatic solvents can be attributed to their aromatic core, which can form *π*–*π* interactions with the conjugated materials.

**Figure 2:**
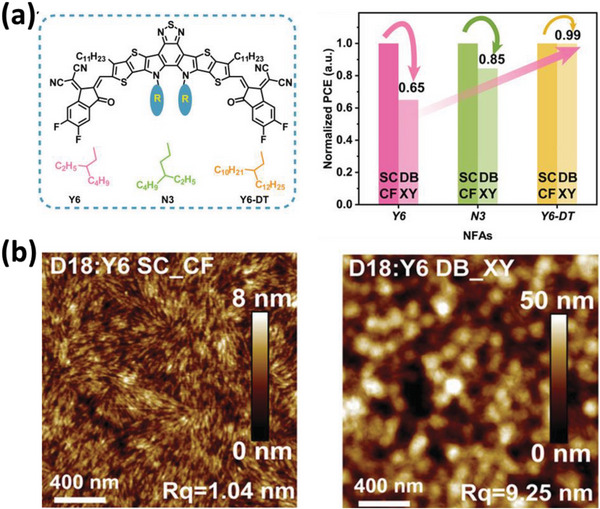
a) The chemical structures of the acceptor Y6 and its derivatives N3 and Y6‐DT with longer side units, and the PCEs difference fabricated from CF and XY based on different acceptors. b) AFM images of the BHJ blends based on D18:Y6 fabricated from CF or XY. Reproduced with permission.^[^ ^29^ ^]^ Copyright 2023, WILEY‐VCH Verlag GmbH & Co.KGaA, Weinheim.

The relatively low *δ*_P_ or *δ*_H_ values of conjugated materials can result in different solubility in halogenated and non‐halogenated aromatic solvents, leading to distinct microstructures. Du et al. observed that the Y6 acceptor has lower solubility in XY than in CF, resulting in strong aggregation of Y6 in XY solution. This aggregation leads to large phase separation in D18:Y6 thin films when being doctor‐bladed from XY, and a lower PCE of 11.08% is finally achieved when compared to the PCE of 16.95% from CF solution (**Figure** **2**).^[^ ^29^ ^]^ The authors also found that elongating the 2‐ethylhexyl side unit to 2‐dodecyltetradecyl side unit (Y6‐DT) improves the solubility of Y6, resulting in comparable PCEs for films fabricated from both CF and XY solutions. This improvement in solubility is likely due to the longer side unit of Y6‐DT.

Li et al. conducted a detailed study of two typical BHJ systems, PM6:BTP‐eC9 and PM6:L8‐BO, which were fabricated from CF, CB, and XY.^[^ ^30^ ^]^ The L8‐BO acceptor with long 2‐butyloctyl (BO) side chains exhibits better solubility in these solvents than that of BTP‐eC9, resulting in L8‐BO in XY having a longer dominated time to form large aggregated domains in BHJ films when solution‐processed from XY. As a result, PM6:L8‐BO blend exhibits a lower PCE of 16.75% fabricated from XY, which is much lower than the PCE of 18.31% fabricated from CF. In contrast, PM6:BTP‐eC9 blends processed with CF and XY show similar PCEs of 17.79% and 18.16%. Further enhancement of PCEs was achieved by adding a third conjugated polymer donor into PM6:BTP‐eC9 blend, providing a PCE of 19.10%, which is also the highest PCEs fabricated from non‐halogenated solvent. The differences in solubility between halogenated and non‐halogenated solvents highlight the importance of optimizing the microstructures of BHJ blends, from the aspect of new design of conjugated materials and optimization of device fabrication, in order to facilitate charge generation and achieve high performance OSCs fabricated from non‐halogenated solvents.

### 3.1 Materials Design Strategy

Poor solubility tends to result in significant aggregation in BHJ thin films processed with non‐halogenated solvents and then solubility enhancement is a potential strategy to address this issue for Y6. One approach to achieving this is to lengthen the alkyl side units of electron acceptors,^[^ ^31^ ^]^ as demonstrated in previous discussion about the work of Du et al. and Li et al.^[^ ^29^, ^30^ ^]^ In addition, Kim et al. examined the impact of outer side chains on the photovoltaic performance of Y‐series acceptors, utilizing XY to fabricate photoactive layers.^[^ ^32^ ^]^ To enhance crystallinity, selenophene was utilized instead of thiophene at the outermost core of Y‐series acceptors, allowing for the use of single‐component XY in the photoactive layer fabrication. Studies demonstrated that the length of the outer side chains (ranging from C_3_H_7_ to C_6_H_13_ and C_9_H_19_) significantly affected the microstructures of the blends. Specifically, PM6:YSe‐C6 blend exhibit the highest PCE of 16.11%. However, extending the side units to C_9_H_19_ leads to a lower PCE of 14.20%.

Additionally, Yip et al. synthesized a fluorinated polythiophene derivative, P4T2F‐HD, and selected Y6‐BO, a Y‐series acceptor with BO side units, for further investigation.^[^ ^33^ ^]^ The goal of the researchers was to increase solubility of both donor and acceptor by employing long side units, enabling them to be soluble in XY. Through Flory–Huggins interaction parameter and differential scanning calorimetry studies, they found that P4T2F‐HD exhibits moderate miscibility with Y6‐BO, whereas P3HT shows good mixing with Y6‐BO. Consequently, a P4T2F‐HD:Y6‐BO blend was solution‐processed from XY with 0.5% diphenylether, leading to a remarkable PCE of 13.34%. This represents the highest PCE of OSCs based on polythiophene derivatives. Furthermore, the simple synthetic procedures resulted in a much improved average figure of merit (aFOM) when compared to other systems, suggesting the great potential for practical applications.

**Figure 3:**
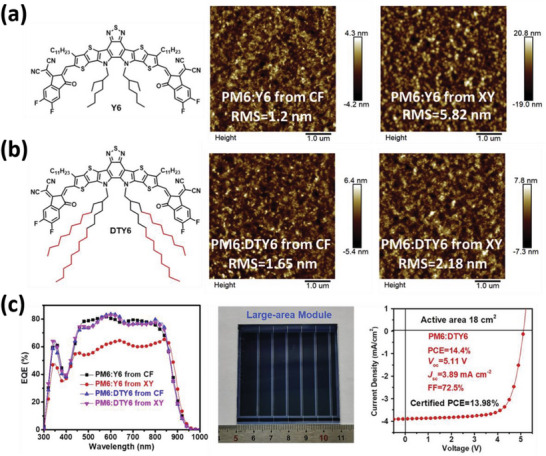
a) The chemical structure of Y6 and the AFM images of PM6:Y6 blends solution‐processed from CF or XY. b) The replacement of Y6 to DTY6 with longer side chains. c) EQE spectra of OSCs fabricated from different solvents, large‐area module and its photovoltaic performance. Reproduced with permission.^[^ ^34^ ^]^ Copyright 2020, Elsevier.

Huang et al. introduced a new non‐fullerene acceptor, DTY6, which contains long‐branched alkyl chains that enable its dissolution in a single‐component non‐halogen solvent for small and large‐area module OSCs. This is illustrated in **Figure** **3**.^[^ ^34^ ^]^ The concept behind the development of DTY6 involves improving the solubility of Y6, a typical acceptor with short alkyl chains, in XY. By introducing long‐branched alkyl chains into Y6, Huang et al. were able to create DTY6, which demonstrated enhanced solubility in XY. Consequently, PM6:DTY6 blends processed from XY exhibited similar phase separation compared to those from chloroform (CF). Notably, the PM6:DTY6 blend could be fabricated from XY solution without the need for high‐boiling additives and displays a high PCE of 16.1% at small‐area devices and 13.98% at 18 cm^2^ devices. These results highlight that introducing long alkyl side chains into conjugated materials is a viable approach to realizing non‐halogenated processed OSCs. It is worthy to mention that, the elongation of alkyl side units is tend to reduce the overall PCEs of OSCs compared to short side units, which has been widely reported in literatures. Therefore, this is not an ideal strategy to realize non‐halogenated processed OSCs.

Another efficient strategy to improve solubility in non‐halogenated solvents is to use asymmetric or random copolymer design. These structures can slightly break the molecular ordering in thin films, thereby enhancing solubility. Such a design approach offers a promising route to overcoming solubility challenges in a range of applications.^[^ ^35^, ^36^, ^37^ ^]^ The strategy of using asymmetric or random copolymer design has been extensively applied in the design of conjugated polymers for non‐halogenated organic field‐effect transistors. This approach can improve both solubility and charge carrier mobilities.^[^ ^38^ ^]^ For instance, Bo et al. incorporated third copolymerized units with a smaller size into the conjugated donor polymers PM6 and D18, resulting in the successful synthesis of polymers PW1 and PW2 with good solubility in 1,2‐dimethylbenzene.^[^ ^39^ ^]^ When BHJ films were processed using this particular solvent, PW1‐ and PW2‐based solar cells achieve higher PCEs of 16.26% and 17.19%, respectively, compared to those based on PM6 and D18 (15.16% and 16.18%).

Other researchers have focused on developing asymmetric Y‐series electron acceptors by introducing different side units at the two N‐positions,^[^ ^40^ ^]^ using two asymmetric end groups at IC position,^[^ ^41^ ^]^ and varying the side units at the TT position.^[^ ^42^ ^]^ All of these designs allow the acceptors to be dissolved in non‐halogenated aromatic solvents, while also enabling the rational tuning of microphase separation in BHJ thin films. As a result, the PCEs of OSCs based on these acceptors, when fabricated using non‐halogenated solvents, can be significantly enhanced.

**Figure 4:**
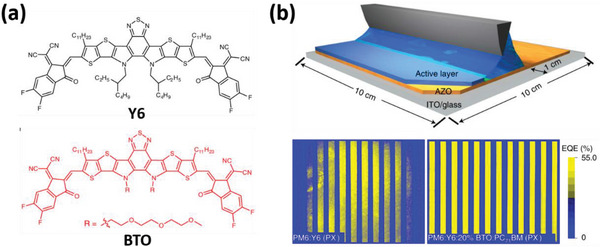
a) The chemical structures of the acceptor Y6 and its derivative BTO with alkyloxy side units. b) Schematic diagram of the blade‐coating process of PX‐processed active layer for large‐area OSC modules and the LBIC mapping images of the blade‐coated OSCs. Reproduced with permission.^[^ ^44^ ^]^ Copyright 2021, Springer Nature Publishing Group.

The third widely reported strategy is to introduce a third component into binary systems to form ternary solar cells. The third component is used to tune the morphology of blends.^[^ ^43^ ^]^ A notable example of this approach was reported by Li et al. in 2021, where they synthesized a third electron acceptor (BTO) with alkyloxy side units to improve the microphase separation of PM6:Y6 blend (see **Figure** **4**).^[^ ^44^ ^]^ When using CB as the solvent, PM6:Y6 blends exhibited large phase separation due to the good solubility of Y6. However, the addition of 20% BTO into the blend significantly lowered the domain size. As a result, PM6:Y6:20% BTO blends yield PCEs of 16.39% and 16.59% when processed from CB and paraxylene (PX), respectively. The researchers then used PX to fabricate 100 cm^2^ modules, and the addition of 20% BTO resulted in homogenous large‐area thin films, as evidenced by the LBIC (light‐beam‐induced current) images. These films produce a high PCE of 14.26%. In comparison, PM6:Y6 blends only show a low PCE of 7.31% at 100 cm^2^ modules.

This strategy has also been reported by other research groups, who have found that the addition of third components can enhance the PCEs of non‐halogenated solvent‐fabricated solar cells.^[^ ^45^, ^46^, ^47^ ^]^ Li et al. employed a quaternary strategy to improve the microstructure of blend thin films. They combined the polymer donor PM6 with two Y‐series acceptors and PC_71_BM, which was then solution‐processed from toluene.^[^ ^46^ ^]^ The resulting quaternary BHJ achieves a PCE of 17.43%. This blend was then used to fabricate semitransparent OSCs. These semitransparent OSCs were found to be beneficial for mung bean sprout growth under greenhouse‐like conditions, due to their visible light transmission properties.

**Figure 5:**
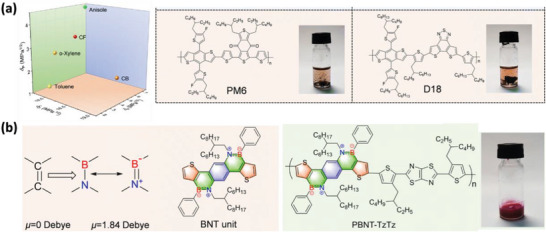
a) The HSP of anisole and other solvents, and the poor solubility of PM6 and D18 in anisole. b) The chemical structures of the donor polymer PBNT‐TzTz, its origination of dipole and solubility in anisole. Reproduced with permission.^[^ ^48^ ^]^ Copyright 2023, The Royal Society of Chemistry.

It is worth noting that most of the donor polymers used for non‐halogenated fabrication are based on PM6 or its derivatives, and only very few studies focus on other polymer donors. For example, Cao et al. reported a B→N based conjugated polymer called PBNT‐TzTz as a donor, which can be dissolved in the food‐grade solvent anisole to make OSCs (**Figure** **5**).^[^ ^48^ ^]^ The good solubility of this B→N conjugated polymer is due to the large dipole moment originating from B→N units, resulting in high polarity that is compatible with anisole's relatively high *δ*_P_. PBNT‐TzTz:L8‐BO‐based solar cells fabricated from anisole with limonene as an additive yield a PCE of 15.65%. This work highlights the effectiveness of rationally adjusting the polarity of conjugated materials to tune their solubility toward environmentally friendly fabrication.

### 3.2 Optimization of Device Fabrication

To improve the microphase separation of bulk heterojunction (BHJ) blends in efficient non‐halogenated/aromatic solution‐processed OSCs, device fabrication optimization is necessary. A commonly used method to achieve this is by adding a high boiling point additive, such as 1,8‐diiodooctane (DIO). However, the addition of DIO can exacerbate the aggregation tendency of Y‐series acceptors due to their low solubility in non‐halogenated solvents.

Interestingly, Zhu et al. recently discovered that blending carbon disulfide (CS_2_) with the Y‐series acceptor (XY) can help to form optimized phase separation in the PM6:Y6 blend. This is because CS_2_ has a relatively low boiling point and good solubility with the Y6 acceptor, which leads to improved microphase separation in the blend.^[^ ^49^ ^]^ OSCs based on PM6:Y6 fabricated from the XY:CS_2_ mixture exhibit a PCE of 16.5%. In contrast, XY‐based OSCs show a significantly lower PCE of only 13.3%. These results demonstrate the importance of optimizing the device fabrication process to achieve efficient OSCs and the potential benefits of using CS_2_ as a blending solvent in BHJ blends.

Li et al. developed a graded bulk heterojunction (BHJ) approach, also known as a pseudo‐bilayer approach, to fabricate efficient OSCs from XY. In this approach, the donor PM6 was first deposited from XY, followed by the deposition of the acceptor BTP‐eC9 from XY with 0.5% DIO to form blend thin films.^[^ ^50^ ^]^ The graded approach facilitated the formation of vertical phase separation in the blend thin film, leading to a high power conversion efficiency (PCE) of 17.48%. Furthermore, the vertical microstructures resulting from the graded BHJ approach ensured efficient charge generation in thick films, as evidenced by the impressive PCE of 14.37% achieved with 500 nm thick active layer. These results show the effectiveness of the graded BHJ approach in improving the microphase separation and device performance of OSCs, particularly those based on XY.

Another strategy to improve the microphase separation of BHJ blends is through hot fabrication method. At high temperature, acceptors exhibit good solubility and less aggregation. Ma et al. conducted a systematic study on the effect of hot slot‐die processing on the photovoltaic performance of OSCs.^[^ ^51^ ^]^ They used PM6:Y6 as the photoactive layer and varied the slot‐die temperature from 80 to 110 °C. The OSCs fabricated from CB, XY, and 1,2,4‐trimethylbenzene exhibit similar power conversion efficiencies (PCEs) of ≈15%, whereas those fabricated at room temperature show significantly lower PCEs ranging from 5.3% to 7.7%. Detailed characterization showed that the improved PCEs achieved with hot‐processing were due to the enhanced crystallinity of the acceptors and the reduced aggregation in BHJ thin films. Li et al. also utilized the hot‐processing method to achieve PCEs over 18% from *p*‐, *o*‐XY, and toluene solutions. These results demonstrate the effectiveness of hot fabrication methods in improving the microphase separation and device performance of OSCs based on various solvents and acceptor materials.^[^ ^52^ ^]^

It is worth mentioning that all‐polymer solar cells using non‐halogenated aromatic solvents based on polymer acceptors that are Stille‐polymerized from Y‐series acceptors have also been widely reported.^[^ ^53^, ^54^, ^55^, ^56^, ^57^, ^58^, ^59^ ^]^ For example, our group selected an all‐polymer blend, PM6:PY‐DT, as the active layer, which could be dissolved in XY.^[^ ^60^ ^]^ By using 2‐methoxynaphthalene as the solid additive, the PM6:PY‐DT blend yields a high PCE of 17.03%, representing the highest PCE based on all‐polymer solar cells processed from a green‐solvent. Cao et al. developed a random copolymerization strategy to synthesize the conjugated donor polymers, in which the electron‐deficient building block from PM6 and dithienobenzothiadiazole (DBT) from D18 were incorporated into one conjugated polymer.^[^ ^61^ ^]^ The results show that the copolymerized donor polymer could reduce the domain size in BHJ thin films fabricated from XY and hence provided a PCE of 17.21% in all‐polymer OSCs.

Non‐halogenated solutions have also found widespread use in the fabrication of devices using blade‐coating or slot‐die coating techniques. One commonly used solvent is XY, owing to its suitable boiling point and solubility to these conjugated materials.^[^ ^44^, ^51^, ^62^, ^63^ ^]^ For example, a dual‐slot‐die processing method for fabricating OSCs devices is reported.^[^ ^62^ ^]^ The slot‐die instrument contained two heads, one for the donor solution (PM6) and the other for the acceptor solution (L8‐BO). During operation, the donor polymer solution was deposited first, followed by the acceptor solution, similar to the pseudo bilayer OSCs. This process resulted in BHJ thin films with a gradient distribution of the donor and acceptor in the vertical direction, which is beneficial for charge transport. This method achieves a PCE of 17.07% for PM6:L8‐BO solar cells fabricated from XY, which is higher than the PCE of 16.49% obtained using traditional mixed solutions.

In summary, most Y‐series based electron acceptors can be dissolved in non‐halogenated/aromatic solvents, making them suitable for use in the fabrication of OSCs. Research has focused on controlling the solubility and crystallization process of these acceptors to achieve optimized microphase separation with small domain size and efficient charge generation, resulting in high PCEs of up to 19%. However, there is a need to pay more attention to the solubility of the donor polymers, which has received less attention. Furthermore, while the donor polymers PM6 and D18 are commonly used, few studies have reported on other donor polymers. Another issue is that many of these non‐halogenated/aromatic solvents‐processed OSCs still use high boiling point additives, such as halogenated 1,8‐diiodooctane, to improve the microstructure of blends, which should also be avoided.

## 4 Non‐Halogenated/Non‐Aromatic Solution‐Processed OSCs

Non‐halogenated and non‐aromatic solvents are considered the best candidates for solution‐processed OSCs. However, poor solubility due to immiscibility has made it difficult to realize this goal. Some studies have found that Y‐series acceptors can be dissolved in tetrahydrogen (THF) and its derivatives, as well as solvents with double bonds (such as limonene), but in most cases, it is not easy to achieve an ideal PCE compared to aromatic solvents. Other studies have focused on alcohol‐solution processed OSCs, where conjugated materials are designed by introducing alkyloxy side units. While these efforts are crucial for realizing completely environmentally friendly OSCs, the PCEs achieved so far remain unsatisfactory.

**Figure 6:**
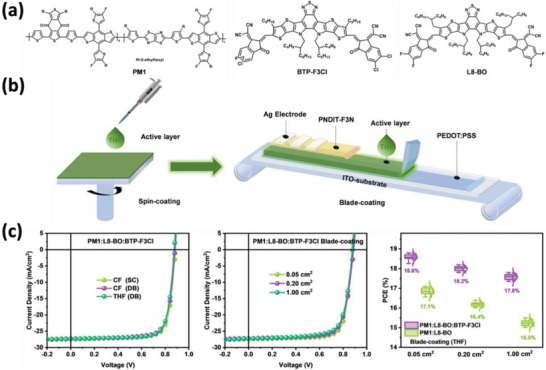
OSCs fabricated from THF solution. a) The chemical structures of the donor polymer and the two acceptors. b) Scheme of doctor‐blade coatings of OSCs by using THF as solvent. c) The *J*–*V* and area‐dependent PCEs. Reproduced with permission.^[^ ^64^ ^]^ Copyright 2022, The Royal Society of Chemistry.

One traditional non‐halogenated and non‐aromatic solvent is THF, which has reasonable HSP (*δ*_D_: 16.8, *δ*_P_: 5.7, *δ*_H_: 8 MPa^1/2^) and can dissolve some conjugated materials. Min et al. reported a ternary OSC system based on PM1 as the donor polymer and L8‐BO:BTP‐3Cl as the alloy acceptors (**Figure** **6**).^[^ ^64^ ^]^ The introduction of BTP‐3Cl improved the absorption spectra, prolonged the exciton lifetime, and enhanced charge transport. The ternary blends could be dissolved in THF and were doctor‐bladed to form thin films. The corresponding OSCs achieve a high PCE of 18.8%, which is comparable to the devices fabricated from CF. Additionally, the 1 cm^2^ OSCs exhibit a PCE of 17.8%, whereas for binary solar cells based on PM1:L8‐BO, the PCE was significantly reduced to 15.5%. These results suggest that the introduction of the third component can alleviate the PCE gap between small and large‐area OSCs. Recently, Woo et al. designed two conjugated polymers as electron donors, in which a benzodithiophene (BDT) segment with fluorothiophene side units was synthesized.^[^ ^65^ ^]^ The position of fluorine atoms on the thiophene units had a significant impact on the microstructure and photovoltaic performance of OSCs. After optimizing the polymer structures and fabricating the photoactive layers from THF, a PCE of 13.86% is achieved, which is comparable to the PCE fabricated from CF.

Since THF shows relatively low boiling point, many works use its derivative 2‐methyltetrahydrofuran (2‐MeTHF) as the solvent to make OSCs. For example, Cao et al. and Huang et al. developed polymer acceptors based on Y‐series monomers and tuned the length of side units to achieve optimized morphology. They fabricated all‐polymer solar cells based on polymer–polymer blends using 2‐MeTHF as the solvent, showing PCEs over 14%.^[^ ^61^, ^66^ ^]^ Welch et al. used 2‐MeTHF as a solvent to fabricate OSCs through slot‐die coating techniques. The OSCs fabricated using all four‐layer slot‐die coating methods achieve a PCE of 9.55%.^[^ ^63^ ^]^

**Figure 7:**
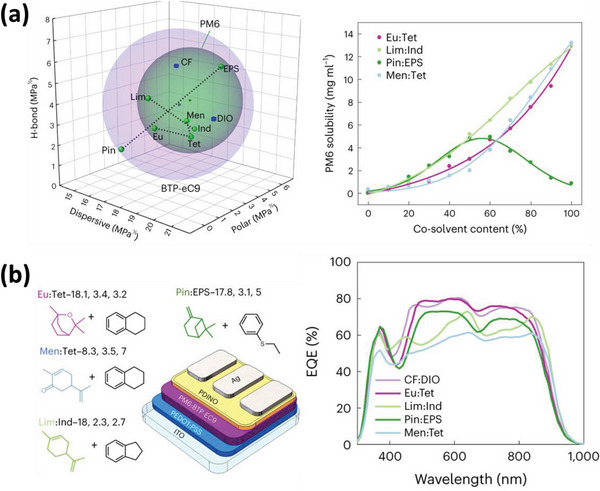
Terpene‐based OSCs. a) 3D representation of the Hansen solubility spheres of PM6 (green sphere) and BTP‐eC9 (pink sphere), and the solubility of PM6 based on terpenes with different content of cosolvents. b) The chemical structures of terpenes, cosolvents, and the configuration of devices, and the EQE spectra of PM6:BTP‐eC9 based OSCs. Reproduced with permission.^[^ ^28^ ^]^ Copyright 2022, Springer Nature Publishing Group.

Terpenes are a class of non‐halogenated and non‐aromatic solvents that have gained attention for their use in fabricating photoactive layers. Limonene, in particular, has been widely studied as a solvent due to its effectiveness in this application.^[^ ^67^, ^68^ ^]^ Baran et al. conducted a comprehensive study on terpene solvents for use in OSCs, which included the use of the binary gradient methodology to determine the HSPs of the donor polymer PM6 and the acceptor BTP‐eC9.^[^ ^28^ ^]^ The HSPiP software was then used to analyze the solubility of different solvents or combination of solvents (**Figure** **7**). Four binary solvent systems were identified that were located within the solubility sphere of both PM6 and BTP‐eC9: eucalyptol:tetraline (Eu:Tet), limonene:indan (Lim:Ind), pinene:ethyl phenyl sulfide (Pin:EPS), and menthone:tetralin (Men:Tet). These four solvent systems were used to fabricate BHJ thin films, and the in situ film formation process was studied in detail. Of the four systems, the Eu:Tet system produces the highest PCE of 15.7%, which is comparable to the PCE of 15.9% achieved using the CF:DIO system. It should be noted that the single terpene solvent is unable to dissolve the donor polymer PM6, requiring the addition of secondary aromatic solvents such as Tet. This work presents a systematic method for selecting potential unknown solvents for OSC fabrication and will undoubtedly inspire new ideas in this field.

**Figure 8:**
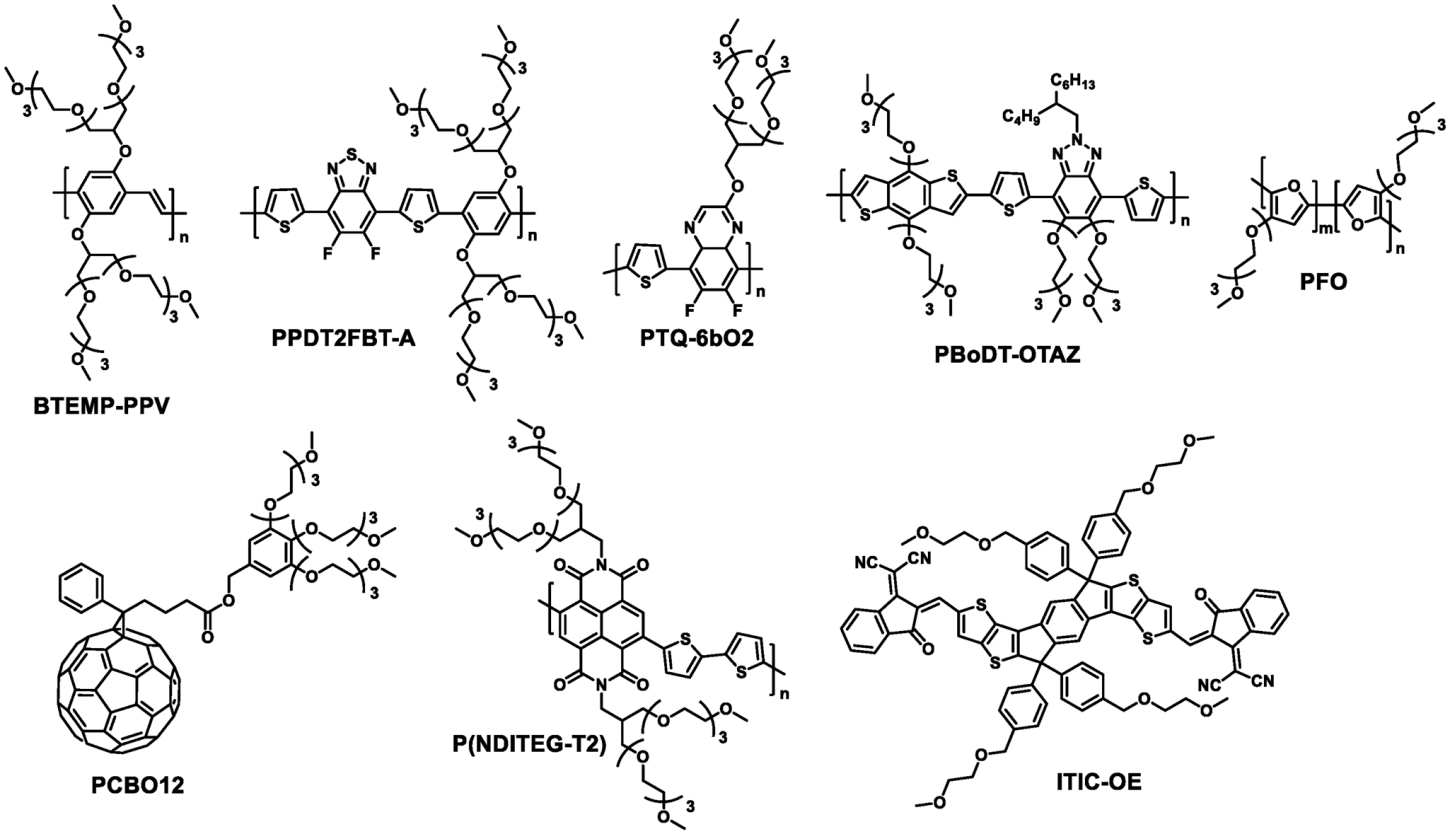
Some representative chemical structures of conjugated materials with alkyloxy side units.

The ultimate goal of OSC fabrication is to use environmentally friendly solvents such as alcohol or water. To achieve this, conjugated materials must be designed with high *δ*_H_ values that are compatible with these solvents. One effective strategy is to introduce alkyloxy side units into conjugated materials, as widely reported in the literature (**Figure** **8**).^[^ ^26^, ^69^, ^70^, ^71^, ^72^, ^73^, ^74^, ^75^, ^76^, ^77^, ^78^, ^79^, ^80^, ^81^ ^]^ Several conjugated donor polymers and electron acceptors, including fullerene, naphthalene diimide‐based polymer acceptors, and ITIC, have been developed with alkyloxy side units and have been used to fabricate OSCs using alcohol and water as the solvents. However, it has been observed that these BHJ systems often exhibit large phase separation, resulting in unsatisfactory PCEs. While a Y‐series acceptor BTO with alkyloxy side units has been synthesized, it still cannot be dissolved in alcohol due to the low content of alkyloxy side units. As a result, there are currently no reports of alcohol or water‐dissolved Y‐series acceptors for use in OSCs. The possible route to design alcohol‐soluble Y‐acceptors is to introduce more alkyloxy side units as other acceptors, but this will reduce the PCEs of OSCs.

In summary, Y‐series acceptors for OSCs can also be processed using non‐halogenated/non‐aromatic solvents, such as THF and its derivatives, and terpenes, achieving PCEs close to 19%. However, the number of studies on this topic is limited compared to those that use non‐halogenated and aromatic solvents. Furthermore, there are currently no Y‐series acceptors that can be dissolved in alcohol and water for OSC fabrication. It is crucial to develop conjugated materials that are compatible with these environmentally friendly solvents and address phase separation issues to achieve more sustainable OSCs. Such advancements could lead to significant improvements in the performance and environmental impact of OSCs.

## 5 Water/Alcohol‐Processed OSCs Based on Conjugated Nanoparticles

Another crucial approach toward environmentally friendly fabrication is to utilize conjugated materials in the form of nanoparticles that can be dispersed in both alcohol and water. The typical procedure for fabricating these nanoparticles involves dissolving the conjugated materials in organic solvents (such as CF or Tol), adding water and surfactant to the solution, dispersing the conjugated materials into a mixture of water and organic solvent using ultrasonic waves, and then removing the organic solvents and surfactant to generate nanoparticles. The performance of these nanoparticles depends on several factors, such as the selection of donor and acceptor materials, the size of the nanoparticles, the phase separation of the donor and acceptor within the nanoparticles, the chemical structures of the surfactants, and the control of the fabrication process. Achieving high performance requires careful consideration of all these factors.^[^ ^82^, ^83^, ^84^, ^85^, ^86^, ^87^, ^88^, ^89^, ^90^, ^91^, ^92^, ^93^, ^94^, ^95^ ^]^

Numerous studies have been conducted on the BHJ systems of P3HT:PCBM or P3HT:ICBA, demonstrating that surfactant‐free nanoparticles can lead to PCEs of around 4% in OSCs^[^ ^84^ ^]^ However, it has been observed that other “donor‐acceptor” conjugated polymers and non‐fullerene acceptors cannot remain stable without surfactants, as they tend to become electrically charged and subsequently repel the nanoparticles.^[^ ^96^ ^]^ While surfactants play a crucial role in the formation of stable nanoparticles, they also possess an insulating nature that can have negative effects on electronic properties.

In order to address this issue, Brabec et al. have investigated various surfactants for dispersing conjugated material nanoparticles and found that poloxamer (Pluronic F127) is capable of stabilizing them.^[^ ^86^ ^]^ Importantly, F127 exhibits a unique characteristic in that its critical micelle concentration is enhanced at high temperatures, indicating that it tends to form loose chains (linear poloxamers) at low temperatures and can be easily removed through centrifugal filtration. By utilizing F127 to disperse conjugated nanoparticles and subsequently removing it, Brabec and colleagues are able to achieve a high PCE of 7.5% in OSCs based on conjugated nanoparticles using this method.

**Figure 9:**
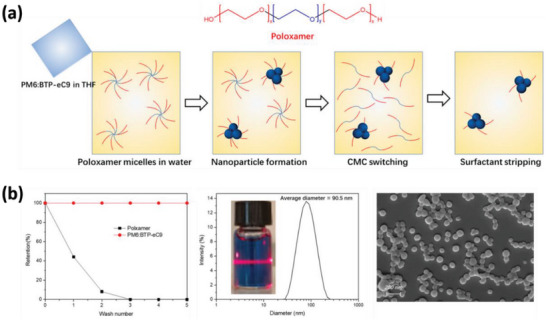
a) Fabrication process of PM6:BTP‐eC9 nanoparticles by using poloxamer as surfactant. b) Characterization of nanoparticles and the retention of poloxamer after several washing times. Reproduced with permission.^[^ ^97^ ^]^ Copyright 2022, The Authors. Published by MDPI.

In a subsequent study, Li and colleagues utilized poloxamer to produce OSCs based on PM6 as the donor and BTP‐eC9 as the acceptor (**Figure** **9**), following the previously described procedure.^[^ ^97^ ^]^ Poloxamer was used initially to disperse the nanoparticles and was then removed via centrifugal filtration at 4 °C. As a result, they were able to obtain a high concentration of 50 mg mL^−1^ conjugated nanoparticles with an average diameter of 90 nm, which was used as the photoactive layer in OSCs. Impressively, they achieve a PCE of 11.1%, which represents the highest PCE reported to date using conjugated nanoparticles. Furthermore, they found that the high boiling point additive DIO played a crucial role in the device performance, as the photoactive layer without the DIO additive achieves a PCE of 9.04%.

Colsmann and colleagues have recently proposed a surfactant‐free method for dispersing conjugated nanoparticles to improve photovoltaic performance.^[^ ^90^ ^]^ Specifically, they added iodine into the CF solution of the J71:Y6 system, which caused rapid precipitation of the solution into acetonitrile to form nanoparticles. Iodine served as an electrical p‐dopant to enhance the colloidal stability of nanoparticle dispersions. Furthermore, iodine's high volatility allowed for its removal after BHJ film formation. The resulting OSCs based on these nanoparticles exhibit a PCE of 10.4%, which surpassed the performance of cells fabricated from CF or CB solutions. However, it is worth noting that the surfactant‐free nanoparticles tend to have relatively low concentrations, which may require multiple spin‐coating steps (e.g., up to 20 steps) to achieve a sufficient thickness (e.g., 90 nm) in the photoactive layers.

As a short summary of these sections, the surfactant or surfactant‐free water/alcohol dispersed conjugated nanoparticles have been developed for application in OSCs. The ink nature of this technique is very promising for industrial application. However, it should also be noted that, the PCEs are still far lagging solution‐processed OSCs. The optimization of synthesis of nanoparticles, from surfactant, solvent, additive, temperature, etc., will be the key to further enhance their photovoltaic performance.

## 6 Conclusion

The recent process for green‐solvent processed non‐fullerene OSCs based on high‐performance Y‐series acceptors has been comprehensively summarized in this study. This includes the use of non‐halogenated/aromatic solvents, non‐halogenated/non‐aromatic solvents, alcohol solvents, and finally water/alcohol dispersed conjugated nanoparticles. The study involved the calculation of HSPs, the innovation of conjugated materials, the optimization of microphase separation, and the fabrication of conjugated nanoparticles, all of which were deeply studied to improve the photovoltaic performance of OSCs. As a result of these efforts, high PCEs exceeding 19% were achieved in XY or THF‐based solution‐processed OSCs, and PCEs exceeding 11% were realized in nanoparticle‐processed OSCs. These techniques were also used to fabricate large‐area devices using doctor‐blade coating or inject printing techniques, showcasing their potential for widespread application. However, some questions and difficulties still need to be addressed in future studies to further advance the field.

Firstly, future studies can focus on conducting more systematic calculations and experiments to determine the HSPs of a wider range of conjugated materials with different conjugated backbones and side units. This will enable the construction of a more comprehensive toolbox for the selection of green solvents and optimization of microphase separation in OSCs. Additionally, investigating the miscibility between different donor and acceptor materials using HSPs can further enhance the efficiency of OSCs.

Secondly, most studies of green‐solvent processed OSCs have focused on toluene or XY‐based aromatic solvents, while non‐aromatic solvents have received less attention. This is partly due to the challenge of identifying suitable solvents to dissolve these conjugated materials. For instance, Baran et al. demonstrated that terpene green solvents can serve as universal solvents for OSCs. However, PM6, a donor polymer, remains insoluble in these solvents, requiring the use of additional aromatic solvents for dissolution.^[^ ^28^ ^]^ To improve the solubility of conjugated materials in non‐halogenated/non‐aromatic solvents, it is necessary to enhance the polarity and hydrogen parameters of these materials. Thus, designing new conjugated materials that incorporate polar units such as alkyloxy units and hydrogen groups is essential. However, the incorporation of these units can also lead to severe aggregation of conjugated materials, resulting in significant phase separation in BHJ thin films. Therefore, new techniques are required to control the morphology in these systems to achieve high performance.

Thirdly, conjugated nanoparticle‐based OSCs are an extremely promising research field for environmentally friendly processing, as they utilize traditional conjugated materials without requiring further structural modification, and solvents such as water and alcohol are completely green. However, their PCEs, which currently sit at 11%, still lag far behind other solution‐processed OSCs. All three photovoltaic parameters, including *V*_oc_, *J*_sc_, and FF, are below traditional solution‐processed methods. Several issues must be addressed, such as nanoparticle size, surfactant species and content, morphology inside the nanoparticles, and the connection between nanoparticles. Choudhury et al. addressed these issues by developing a sulfonated thiophene derivative as a surfactant. The conjugated thiophene can act as a building block to connect the donor and acceptor and improve blend morphology in nanoparticles.^[^ ^98^ ^]^ Such designs could help balance the detrimental effects of surfactants and their dispersion function. We anticipate that conjugated nanoparticle‐based OSCs will be one of the most promising techniques for industrial application and should receive more attention.

Lastly, the majority of studies focus on environmentally friendly solution processes for BHJ thin films, but very few studies address the concern of using green procedures for the synthesis of conjugated materials. Although some synthetic methods, such as direct (hetero)arylation polymerization,^[^ ^99^ ^]^ have been utilized to construct conjugated materials, their photovoltaic performance still lags behind conjugated materials produced through traditional synthetic methods, such as Stille polymerization. The low reactivity and defects still hinder the development of new synthetic methods for conjugated materials. Therefore, it is necessary to explore new catalysts and monomers to improve reactivity in these green synthetic methods.

In summary, OSCs based on Y‐series electron acceptors can be solution‐processed from green solvents with high PCEs. However, the studies are still at an early stage, particularly for conjugated nanoparticle‐based OSCs. Further efforts are necessary to improve the solution fabrication process and photovoltaic performance, including the development of new conjugated materials and processing methods. These efforts will enable OSCs to enter large‐scale production and find practical applications in our daily lives.

## Conflict of Interest

The authors declare no conflict of interest.
